# Polymorphisms in the glucocorticoid receptor gene that modulate glucocorticoid sensitivity are associated with rheumatoid arthritis

**DOI:** 10.1186/ar3118

**Published:** 2010-08-21

**Authors:** Manon JM van Oosten, Radboud JEM Dolhain, Jan W Koper, Elisabeth FC van Rossum, Marieke Emonts, Khik H Han, Jacques MGW Wouters, Johanne MW Hazes, Steven WJ Lamberts, Richard A Feelders

**Affiliations:** 1 Department of Internal Medicine, Section of Endocrinology, Erasmus MC, 's-Gravendijkwal 230, 3015 CE, Rotterdam, The Netherlands; 2 Department of Rheumatology, Erasmus MC, 's-Gravendijkwal 230, 3015 CE, Rotterdam, The Netherlands; 3 Department of Pediatrics and Department of Immunology, Erasmus MC-Sophia, Dr. Molewaterplein 60, 3015 GJ, Rotterdam, The Netherlands; 4 Department of Rheumatology, Maasstad Hospital, Olympiaweg 350, 3078 HT, Rotterdam, The Netherlands; 5 Department of Rheumatology, Sint Franciscus Hospital, Kleiweg 500, 3045 PM, Rotterdam, The Netherlands

## Abstract

**Introduction:**

The glucocorticoid receptor (GR) plays an important regulatory role in the immune system. Four polymorphisms in the GR gene are associated with differences in glucocorticoid (GC) sensitivity; the minor alleles of the polymorphisms N363 S and *Bcl*I are associated with relative hypersensitivity to GCs, while those of the polymorphisms ER22/23EK and 9*β *are associated with relative GC resistance. Because differences in GC sensitivity may influence immune effector functions, we examined whether these polymorphisms are associated with the susceptibility to develop Rheumatoid Arthritis (RA) and RA disease severity.

**Methods:**

The presence of GR polymorphisms was assessed in healthy controls (*n *= 5033), and in RA patients (*n *= 368). A second control group (*n *= 532) was used for confirmation of results. In RA patients, the relationship between GR polymorphisms and disease severity was examined.

**Results:**

Carriers of the N363 S and *Bcl*I minor alleles had a lower risk of developing RA: odds ratio (OR) = 0.55 (95% confidence interval (CI) 0.32-0.96, *P *= 0.032) and OR = 0.73 (95% CI 0.58-0.91, *P *= 0.006), respectively. In contrast, 9*β *minor allele carriers had a higher risk of developing RA: OR = 1.26 (95% CI 1.00-1.60, *P *= 0.050). For ER22/23EK minor allele carriers a trend to an increased risk OR = 1.42 (95% CI 0.95-2.13, *P *= 0.086) was found. All ER22/23EK carriers (32/32) had erosive disease, while only 77% (259/336) of the non-carriers did (*P *= 0.008). In addition, ER22/23EK carriers were treated more frequently with anti-tumor necrosis factor-alpha (TNF*α*) therapy (*P *< 0.05).

**Conclusions:**

The minor alleles of the 9*β *and ER22/23EK polymorphisms seem to be associated with increased predisposition to develop RA. Conversely, the minor alleles of the N363 S and *Bcl*I polymorphisms are associated with reduced susceptibility to develop RA. These opposite associations suggest that constitutionally determined GC resistance may predispose to development of auto-immunity, at least in RA, and vice versa.

## Introduction

Rheumatoid arthritis (RA) is a chronic inflammatory disease in which dysregulation of the immune system plays a central role. However, some parts of its etiology remain largely unknown. Several lines of evidence suggest that dysregulation of the hypothalamic-pituitary-adrenal (HPA) axis may play a contributory role in the pathogenesis of RA [1]. In Lewis rats, used as animal model for chronic arthritis, an impaired HPA-axis activity is clearly associated with the development of chronic, erosive arthritis, which can be antagonized by administration of dexamethasone [2]. In patients with RA, plasma adrenocorticotropic hormone and cortisol levels are relatively low despite the presence of severe inflammation and high IL-6 levels, a major HPA-axis stimulus during inflammation [3]. In addition, the number of glucocorticoid receptors (GRs) in RA patients was significantly lower than in controls and the cortisol response to surgical stress is attenuated in patients with RA [4,5]. These observations suggest that a blunted HPA-axis is a contributory factor in the development and progression of RA.

Apart from the glucocorticoid (GC) concentration, the ultimate biological effects of GCs are also determined by an individual's GC sensitivity. GCs exert their effects by binding to the GR. Population studies identified several polymorphisms in the GR gene, on chromosome 5, which are associated with differences in GC sensitivity [6,7]. The minor alleles of the GR polymorphisms N363 S (rs6195) and *Bcl*I (rs41423247) are associated with a relative hypersensitivity to GCs. The N363 S polymorphism (identified in codon 363 of exon 2) is located in the part of the GR gene that encodes the GR transactivating domain [6]. The *Bcl*I polymorphism is an intronic restriction fragment length polymorphism located 646 bp downstream from exon 2 [8]. Carriers of the minor alleles of the N363 S and *Bcl*I polymorphisms have lower cortisol levels after administration of low-dose dexamethasone compared with non-carriers of this allele and have phenotypic features reminiscent of endogenous hypercortisolism such as an increased body mass index, abdominal obesity, increased insulin response and depression [8,9].

In contrast, the minor alleles of the ER22/23EK (rs6189 and rs6190) and 9*β *(rs6198) polymorphisms are both associated with a relative GC resistance [10,11]. The ER22/23EK polymorphism is also located in the part of the GR gene that encodes the transactivating domain and consists of two linked single nucleotide mutations in codons 22 and 23 in exon 2 [6]. The 9*β *polymorphism is located in exon 9*β *and is thought to increase the stability of the GR splice variant GR*β*, which acts as a dominant inhibitor of the functional wild-type GR*α *[12]. Carriers of the minor alleles of the ER22/23EK polymorphism show a decreased response to the administration of 1 mg dexamethasone [7,10]. Furthermore, carriers of the minor alleles of ER22/23EK tend to have a beneficial metabolic profile, reflected by significantly lower fasting insulin levels, lower cholesterol levels, and lower high-sensitive C-reactive protein levels, as well as a favorable body composition and decreased risk of dementia [13-15]. The GR polymorphism 9*β *was found to be associated with RA in a small group of patients, as well as with a reduced risk for *Staphylococcus aureus *nasal carriage in healthy subjects, which may indicate reduced GC-induced immunosuppression [16,17].

As GR polymorphisms may modulate GC responsiveness of the immune system, we hypothesize that polymorphisms in the GR gene, which are accompanied by relative GC hyper- and hyposensitivity, are associated with a decreased or increased susceptibility to develop RA, respectively. To further explore possible involvement of genetically determined GC sensitivity in RA, we examined the prevalence of these polymorphisms as well as the relation with disease severity in a well-defined population of patients with severe RA and controls.

## Materials and methods

### Participants

Three-hundred and sixty-eight Caucasian patients who met the American College of Rheumatism 1987 criteria [18] were included in the study. Additionally, all patients had to be either rheumatoid factor positive, anti-cyclic citrullinated peptide (CCP) positive, or have joint erosions.

Radiographic assessment of the joints was used to estimate the presence of joint erosion consistent with RA. In 97% of the RA patients (*n *= 357) radiographic data were available. Anti-CCP test data were available in 34% of patient (*n *= 125).

The use of anti-TNF*α *therapy was used as a marker of disease severity. In the Netherlands this therapy is only prescribed and reimbursed by health insurance companies for RA patients with therapy-resistant disease. Therapy-resistance is defined as failure of at least two disease-modifying anti-rheumatic drugs (DMARDs), including methotrexate, and still active disease (defined as disease activity score using 28 joint counts > 3.2) despite therapy with methotrexate 25 mg weekly or with methotrexate at the maximum tolerated dose. Approximately 32% of the RA patients (*n *= 117) were treated with anti-TNF*α *therapy.

The study was approved by the local ethics committee. Written informed consent was obtained from all participants.

### Controls

Participants of the Rotterdam Study were used as a control group. The Rotterdam Study is a (Caucasian) population-based cohort study, designed to study the frequency and determinants of chronic diseases in the elderly. All inhabitants of Ommoord, a suburb of Rotterdam, the Netherlands, aged 55 years and over were invited, of whom 7,983 participated in this study [19]. Participants were excluded from this control group if they reported to be diagnosed with RA, or reported the use of anti-inflammatory and anti-rheumatic drugs or immunomodulating agents. A total of 5,033 participants, who met the inclusion criteria and were completely genotyped, was included in the control group.

A second control group consisting of 532 healthy volunteer blood donors, of whom only age and sex were known, was used for comparison and confirmation of the results.

### DNA extraction and genotyping

On enrollment in the study, blood was drawn from each patient and DNA was extracted from fresh blood following standard procedures. All participants were genotyped for the functional GR polymorphisms N363 S (rs6195), *Bcl*I (rs41423247), ER22/23EK (rs6189 and rs6190) and 9*β *(rs6198). Genotyping was performed using the allelic discrimination technique, with custom designed primers and probes (Assay by Design service, Applied Biosystems, Nieuwerkerk aan den IJssel, The Netherlands, primer and probe sequences available on request), using TaqMan Universal PCR master mix (Applied Biosystems, Nieuwerkerk aan den IJssel, The Netherlands). Reaction components and amplification parameters were based on the manufacturer's instructions.

### Statistical analysis

All analyses with regard to the differences between cases and controls were performed using a binary logistic regression model. Crude odd ratios (OR), as estimates of the relative risks, were calculated with 95% confidence intervals (CI), and accordingly expressed with lower and upper bounds. For all four polymorphisms, ORs were calculated from the proportion of minor allele carriers in cases and controls. Additionally, for the *Bcl*l and 9*β *polymorphisms, ORs were computed for homozygous and heterozygous carriers of the minor alleles, separately. This subdivision was not applied to the ER22/23EK and N363 S carriers because of their lower prevalence. The computations were repeated with adjustment for sex and age, by implementing the variables in the model.

Analyses for binary disease parameters, that is anti-TNF*α *therapy use, medication use, and radiographic joint erosion, were performed using a binary logistic regression model. In case of a continuous disease parameter, that is number of medications per disease year, a general linear model was used. These analyses were also performed with adjustment for age and sex by multivariable modelling.

The presented *P *values are two-sided throughout, and a *P *< 0.05 was considered statistically significant. Data were analyzed using SPSS for Windows, release 12.0.1 (SPSS, Chicago, IL, USA).

## Results

### Patients and controls

For 368 RA patients, a complete data set on disease parameters, confounding variables and GR genotypes was available. Two control groups were studied, the first consisted of 5,033 participants of the Rotterdam study, and the second group consisted of 532 healthy blood donors. Table 1 presents the baseline characteristics for case and control groups. Both age and sex were significantly different between the groups (*P *< 0.01). Rheumatoid factor was present in 91% of the RA patients. Anti-CCP was present in 83% of the RA patients who were tested. The median number of DMARDs used per disease year was three. Anti-TNF*α *compounds were administered to 117 of 364 patients.

**Table 1:** Baseline characteristics of the RA patients population and two control populations

	RA *n *= 368	Rotterdam study *n *= 5033	Blood donors *n *= 532
Mean age (SD)	59.5 (15.2)	68.7 (8.8)	42.7 (7.8)
Gender (% female)	73.4	57.6	45.1
			
Median disease duration	8.5 (0-54)		
Anti-CCP positive*	104/125 (83.2%)		
RF positive	336/367 (91.6%)		
Erosion	291/357 (81.5%)		
Median nr DMARDs	3 (1-13)		
Anti-TNF*α *use	117/364 (32%)		

* anti-CCP was not routinely analysed.

CCP, cyclic citrullinated peptide; DMARDs, disease-modifying anti-rheumatic drugs; RA, rheumatoid arthritis; RF, rheumatoid factor; SD, standard deviation; TNF, tumor necrosis factor.

Genotype frequencies for all polymorphisms in both control groups were in Hardy-Weinberg Equilibrium. The *Bcl*I and 9*β *carriers are subdivided into heterozygous and homozygous carriers. Between both control groups, no significant differences were found in the prevalence of all four polymorphisms.

### Case-control associations

The prevalence of GR polymorphisms was assessed in RA patients and compared with the prevalence among participants of the Rotterdam study. After adjustment for the possible confounding factors age and sex, three out of four polymorphisms showed a significant association with RA (Table 2). Carriers of the N363 S and *Bcl*I minor alleles had a lower risk of developing RA: OR = 0.55 (95% CI = 0.32 to 0.96, *P *= 0.034) and OR = 0.73 (95% CI = 0.58 to 0.91, *P *= 0.006), respectively. In contrast, the minor allele of the 9*β *polymorphism was associated with an increased risk of developing RA: OR = 1.26 (95% CI = 1.00 to 1.60, *P *= 0.05). For ER22/23EK minor allele carriers also an increased risk OR = 1.42 (95% CI = 0.95 to 2.13, *P *= 0.086) was found, although not significant.

**Table 2:** Frequencies of GR polymorphisms in RA patients vs. controls from the Rotterdam study, adjusted for age and sex

Polymorphism	RA Case, n (%)	Rotterdam study Control, n (%)	Unadjusted OR (95% CI)	*P*-value	Adjusted OR (95% CI)	*P*-value
ER22/23EK						
Non-carriers	336 (91.3%)	4721 (93.8%)	reference		reference	
Carriers	32 (8.7%)^1^	312 (6.2%)^2^	1.44 (0.99-2.10)	0.058	1.42 (0.95-2.13)	0.086
N363S						
Non-carriers	352 (95.7%)	4664 (92.7%)	reference		reference	
Carriers	16 (4.3%)	369 (7.4%)^3^	0.58 (0.35-0.95)	0.032	0.55 (0.32-0.96)	0.034
*Bcl*I						
Non-carriers	176 (47.8%)	1979 (39.3%)	reference		reference	
Carriers*	192 (52.2%)	3054 (60.7%)	0.71 (0.57-0.87)	0.001	0.73 (0.58-0.91)	0.006
Heterozygous carriers	149 (40.5%)	2370 (47.1%)	0.71 (0.56-0.89)	0.003	0.72 (0.57-0.92)	0.008
Homozygous carriers	43 (11.7%)	684 (13.6%)	0.71 (0.50-0.996)	0.047	0.74 (0.52-1.07)	0.108
9β						
Non-carriers	233 (63.3%)	3445 (68.4%)	reference		reference	
Carriers*	135 (36.7%)	1588 (31.6%)	1.26 (1.01-1.57)	0.041	1.26 (1.00-1.60)	0.050
Heterozygous carriers	124 (33.7%)	1437 (28.6%)	1.28 (1.02-1.60)	0.035	1.13 (1.004-1.62)	0.047
Homozygous carriers	11 (3.0%)	151 (3.0%)	1.08 (0.58-2.00)	0.816	1.08 (0.58-2.22)	0.720

^1 ^One homozygous carrier.

^2 ^Eight homozygous carriers.

^3 ^Four homozygous carriers.

* This includes hetero- and homozygous carriers.

CI, confidence interval; GR, glucocorticoid receptor; OR, odds ratio; RA, rheumatoid arthritis.

The outcomes were verified by comparing the prevalence of GR polymorphisms in the group of RA patients, with the second control group of healthy blood donors (Table 3). Once again *Bcl*I carriers showed a lower risk of developing RA (OR = 0.61, 95% CI = 0.43 to 0.86, *P *= 0.005), as did the N363 S carriers (OR = 0.71, 95% CI = 0.34 to 1.50, *P *= 0.380). And conversely, carriers of the 9*β *polymorphism demonstrated an increased risk of developing RA (OR = 1.44, 95% CI = 1.00 to 2.07, *P *= 0.049), just like the ER22/23EK carriers (OR = 1.54, 95% CI = 0.82 to 2.90, *P *= 0.181). These results are consistent with the effects found when compared with the first control group.

**Table 3:** Frequencies of GR polymorphisms in RA patients vs. blood donor controls, adjusted for age and sex

Polymorphism	RA Case, n (%)	Blood donors Control, n (%)	Unadjusted OR (95% CI)	*P*-value	Adjusted OR (95% CI)	*P*-value
ER22/23EK						
Non-carriers	336 (91.3%)	495 (93.0%)	reference		reference	
Carriers	32 (8.7%)^1^	37 (7.0%)^2^	1.27 (0.78-2.08)	0.335	1.54 (0.82-2.90)	0.181
N363S						
Non-carriers	352 (95.7%)	493 (92.7%)	reference		reference	
Carriers	16 (4.3%)	39 (7.3%)	0.58 (0.32-1.04)	0.066	0.71 (0.34-1.50)	0.380
*Bcl*I						
Non-carriers	176 (47.8%)	199 (37.4%)	reference		reference	
Carriers*	192 (52.2%)	333 (62.6%)	0.65 (0.50-0.85)	0.002	0.61 (0.43-0.86)	0.005
Heterozygous carriers	149 (40.5%)	255 (47.9%)	0.66 (0.50-0.88)	0.004	0.63 (0.44-0.92)	0.015
Homozygous carriers	43 (11.7%)	78 (14.7%)	0.62 (0.41-0.95)	0.028	0.53 (0.31-0.92)	0.024
9β						
Non-carriers	233 (63.3%)	367 (69.0%)	reference		reference	
Carriers*	135 (36.7%)	165 (31.0%)	1.29 (0.97-1.71)	0.076	1.44 (1.001-2.07)	0.049
Heterozygous carriers	124 (33.7%)	147 (27.6%)	1.33 (0.995-1.78)	0.054	1.52 (1.05-2.22)	0.028
Homozygous carriers	11 (3.0%)	18 (3.4%)	0.96 (0.45-2.04)	0.992	0.85 (0.32-2.27)	0.746

^1 ^One homozygous carrier.

^2 ^One homozygous carrier.

* This includes hetero- and homozygous carriers.

CI, confidence interval; GR, glucocorticoid receptor; OR, odds ratio; RA, rheumatoid arthritis.

**Figure 1:**
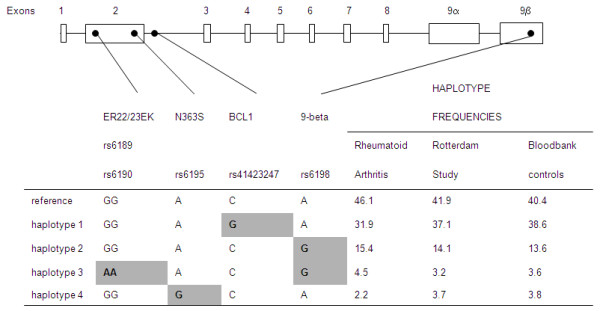
**Polymorphisms in the glucocorticoid receptor gene and the deduced haplotypes**. Position of the polymorphisms are indicated in relation to the gene (top, not drawn to scale) and the sequence changes and the haplotypes and their frequencies are shown (bottom).

Using the program Phase (version 2.1, Matthew Stephens Lab, University of Chicago, Chicago, IL, USA), we reconstructed the haplotypes for these populations (illustrated in Figure 1); however, analysis with these haplotypes did not result in different ORs (data not shown) [20,21].

### Severity of disease

We used three different parameters to assess the severity of disease, namely erosive disease, the use of anti-TNF*α *therapy and number of medications used per disease year.

ER22/23EK carriers were treated more frequently with anti-TNF*α *therapy: OR = 2.90 (95% CI = 1.08 to 7.80, *P *= 0.035). None of the other polymorphisms showed a significant association with erosive disease or anti-TNF*α *therapy. All ER22/23EK carriers (32 of 32) had erosive disease, whereas only 77% (259/336) of the non-carriers did (*P *= 0.008). However, it should be noted that in some patients X-rays were performed only at diagnosis and it is likely that in patients who have more severe disease, X-rays were performed more often, increasing the chance of finding erosion.

To assess the number of medications per disease year we selected patients who had a disease duration of more than five years. This was performed to correct for the fact that at present it is more common to treat newly diagnosed patients with initial combination therapy and to change DMARDs more frequently in case of inadequate response. This can distort a possible association between the number of medications and the GR polymorphisms. A total of 257 patients was eligible to assess the association between GR polymorphisms and number of medications per disease year, but no significant association with any of the GR polymorphisms was found.

## Discussion

In this study we examined whether functional polymorphisms of the GR gene are associated with RA susceptibility. We show that carriers of GR polymorphisms associated with increased GC sensitivity (the minor alleles of the N363 S and *Bcl*I polymorphisms) have a lower risk of developing RA, in contrast to carriers of GR polymorphisms associated with relative GC resistance (the minor alleles of the ER22/23EK and 9β polymorphisms) who were overrepresented among RA patients. These opposite associations may point to a contributory role for GC sensitivity in the susceptibility to develop RA.

The pathogenesis of synovial inflammation in RA is complex and involves development of autoreactivity with an important role for T-cell activation, auto-antibodies, proinflammatory cytokines and specific HLA-DR genes as genetic predisposing factors [1]. Apart from enhanced proinflammatory pathways, attenuation of anti-inflammatory mechanisms may play a contributory role in the development of auto-immunity. Defects in the HPA-axis with a concomitant decreased endogenous immunosuppression have been implicated in RA [2,3,5].

In addition to a relatively low cortisol concentration, changes in GC sensitivity could be involved in the pathogenesis of RA. GCs act via the GR and after binding of GC this ligand-bound complex translocates into the nucleus where it can either bind to GC responsive elements or directly to transcription factors. This results in upregulation ('transactivation') or downregulation ('transrepression') of target gene transcription. Immunosuppressive effects of GCs are mainly mediated by direct protein-protein interaction where the GR functions as a transcription factor leading to transrepression of genes encoding for, for example, cytokines and proinflammatory transcription factors [22]. GC sensitivity is determined by both acquired and genetic factors. Acquired factors include disease-related factors such as cytokines, which are known to induce GC resistance in inflammatory areas, whereas genetic factors involve functional GR polymorphisms that modulate GC sensitivity, as well as other variants that may affect the activity of the HPA axis [23].

In this study we explored the involvement of genetically determined GC sensitivity in RA. Frequencies of the minor alleles of the GR polymorphisms that are associated with GC hypersensitivity *(BcI*I and N363 S, 7-9) were significantly lower in RA patients compared with healthy controls, which indeed might imply that carriers of these polymorphisms are less prone to develop RA. In contrast, frequencies of the minor alleles of GR polymorphisms associated with a relative resistance to GCs (ER22/23EK and 9β, 10,11,16,17) were substantially higher in the RA group compared with healthy controls. However, the results for ER22/23EK carriers were not significant at the employed significance level of 5%, which may be due to the low frequency of this polymorphism in the general population. When a one-sided test was used, which could be appropriate because we assume that relative GC resistance leads to an increased susceptibility to RA, the results were significant. On the other hand, it is difficult to assess the influence of the 9β polymorphism (which is always present in combination with ER22/23EK, see Figure 1 haplotype 3) in this case.

In this study significant differences were present in mean age and gender ratio between the RA patients and controls. These can lead to a confounding bias, causing an over- or underestimation of the effects in question. However, based on what is currently known about the relation between age and sex and GR polymorphisms, it is unlikely that these differences are relevant with regard to the observed frequencies of GR polymorphisms. Only for the ER22/23EK polymorphism, sex-related and age-related differences have been reported. In young adults this polymorphism appeared to be associated with sex-specific differences in body composition and in elderly men carriers of the ER22/23EK polymorphism had a better survival compared with non-carriers [13,14]. Yet, to control for the possible role of confounding bias in this study, we adjusted all analyses for age and sex by multivariable modeling. In addition, a second control group consisting of healthy blood donors was used for comparison of results. Adjustment for both age and sex gave no significantly different results in any of the GR polymorphisms. Furthermore, frequencies of the four GR polymorphisms were comparable in the two control groups, which differed significantly in mean age (Table 1). Based on these findings we conclude that the observed associations between the four investigated GR polymorphisms and RA are unbiased with respect to age and sex.

With respect to the 9*β *polymorphism our results are a confirmation of the findings of DeRijk and colleagues who studied the prevalence of this polymorphism in a small group of 30 RA patients [16]. They showed that there were significantly more 9*β *carriers in the group of RA patients compared with a control group. Lee and colleagues studied the prevalence of the GR polymorphism *Bcl*I in a group of 149 Korean RA patients, but found no significant difference between RA patients and controls [24]. It should be noted that this population is of different ethnic origin and can thus not be compared with our study population. Recently, Donn and colleagues found no association between RA and the GR polymorphisms N363 S, *Bcl*I ER22/23EK or 9*β *in 198 Caucasian RA patients [25]. This might be explained by a lack of statistical power. In particular to investigate the polymorphisms with a low prevalence (N363 S and ER22/23EK) the sample size of this study is relatively small. The pathogenesis of RA is a result of a complex interaction between environmental and several genetic factors. For that reason the contribution of individual genetic factors is relatively low and therefore genetic studies on RA should have enough power to detect even small ORs [26]. In addition, the studied patient population in the article by Donn and colleagues may not be comparable with our study population. No data were provided on the presence or absence of rheumatoid factor nor on treatment characteristics, that is the type and amount of anti-rheumatic medication. The strength of our patient group is that it is a large homogenous cohort of patients with severe RA, as can be appreciated from the fact that more than 90% of patients are rheumatoid factor positive and approximately one third of patients used anti-TNF*α *therapy. As the general RA patient population is heterogeneous with respect to degree of inflammation, the observed association between RA and GR polymorphisms may only apply to a subgroup consisting of RA patients with severe disease activity.

Apart from the association between the polymorphisms with susceptibility of disease we also investigated whether relative GC resistance could possibly lead to an earlier onset of RA and/or a more aggressive disease phenotype. Severity of disease was indicated by radiographic joint erosion, number of DMARDs used and the administration of anti-TNF*α *therapy. Carriers of the ER22/23EK polymorphism showed significantly more joint erosion and were treated more frequently with anti-TNF*α *therapy, which suggests that a relative GC resistant state is related to a more severe disease phenotype.

In our patient cohort no data were available on treatment response after GC therapy. It might be hypothesized that GR polymorphisms influence the clinical response to GC treatment, which could be examined in future studies.

## Conclusions

In conclusion, the GC-resistant polymorphisms ER22/23EK and 9*β *are associated with an enhanced predisposition to develop RA. Conversely, GC hypersensitive polymorphisms N363 S and *Bcl*I are associated with a reduced susceptibility to develop RA. These opposite associations suggest that genetically determined GC sensitivity might play a role in the development of auto-immunity, at least in RA. In addition, relative GC resistance, as in the ER22/23EK polymorphism, may lead to a more aggressive disease phenotype.

## Abbreviations

bp: base pair; CCP: cyclic citrullinated peptide; CI: confidence interval; DMARDs: disease-modifying anti-rheumatic drugs; GC: glucocorticoid; GR: glucocorticoid receptor; HPA-axis: hypothalamic-pituitary-adrenal axis; OR: odds ratio; RA: rheumatoid arthritis; TNF: tumor necrosis factor.

## Competing interests

The authors declare that they have no competing interests.

## Authors' contributions

MJMvO carried out the laboratory work, the statistical analysis and wrote the paper. RJEMD participated in co-writing the paper and research supervision. JWK participated in the laboratory work, co-writing the paper and research supervision. EFCvR participated in the statistical analysis of the paper. ME, KHH and JMGWW participated in design of the study and collection of patient data. JMWH and SWJL participated in research supervsion. RAF participated in co-writing the paper and research supervision. All authors read and approved the final manuscript.
